# Study of the Relationship between Changes in the Structural, Optical, and Strength Properties of AlN Ceramics Subjected to Irradiation with Heavy Xe^23+^ Ions

**DOI:** 10.3390/ma16196362

**Published:** 2023-09-22

**Authors:** Yeugeniy V. Bikhert, Artem L. Kozlovskiy, Anatoli I. Popov, Maxim V. Zdorovets

**Affiliations:** 1 Engineering Profile Laboratory, L.N. Gumilyov Eurasian National University, Satpaev Str. 5, Astana 010008, Kazakhstan; kozlovskiy.a@inp.kz (A.L.K.); mzdorovets@gmail.com (M.V.Z.); 2 Laboratory of Solid State Physics, The Institute of Nuclear Physics, Almaty 050032, Kazakhstan; 3 Institute of Geology and Oil and Gas Business, Satbayev University, Almaty 050032, Kazakhstan; 4 Institute of Solid State Physics, University of Latvia, 8 Kengaraga Str., LV-1063 Riga, Latvia; popov@latnet.lv

**Keywords:** radiation damage, structural changes, deformations, heavy ions, nitride ceramics, construction materials

## Abstract

The purpose of this study is to comprehensively analyze the influence of different fluences of irradiation with Xe^23+^ heavy ions on alterations in the structural, optical, and strength properties of AlN ceramics and to establish a connection between structural distortions and alterations in the optical and mechanical properties of the ceramics. X-ray diffraction, UV-Vis and Raman spectroscopy, and indentation and single-compression methods were used as research methods. During the study, it was demonstrated that at low irradiation fluences, the main role in the changes in the properties of the AlN ceramics is played by effects related to changes in their optical properties and a fundamental absorption edge shift, which characterizes changes in the electronic properties of the ceramics (changes in the distribution of electron density). A study of the variations in the optical properties of the examined samples in relation to the irradiation fluence showed that when the fluence surpasses 5 × 10^11^ ion/cm^2^, an extra-spectral absorption band emerges within the range of 3.38–3.40 eV. This band is distinctive for the creation of vacancy *O_N_*–*V_Al_* complexes within the damaged layer’s structure. The presence of these complexes signifies structural deformations and the accumulation of defective inclusions within the damaged layer. An analysis of changes in the parameters of the crystal lattice showed that structural distortions in the damaged layer are due to the accumulation of tensile residual mechanical stresses, an increase in the concentration of which leads to the swelling and destruction of the damaged layer. Some correlations between the mechanical properties of ceramics and the irradiation fluence indicate the ceramics’ remarkable resistance to radiation-induced brittleness and weakening. These effects become apparent only when structural damage accumulates, resulting in the swelling of the crystal lattice exceeding 2.5–3%.

## 1. Introduction

### 1.1. Prospects for Ceramics in Nuclear Power

In light of recent trends in the world, considerable focus and effort have been dedicated to the advancement of alternative energy resources, the main purpose of which, in addition to solving a number of environmental problems associated with pollution and carbon dioxide emissions, is to reduce the dependence of the energy sector on hydrocarbons [1,2]. A large stake in this area of research is placed on nuclear and thermonuclear energy, increases in the capacities of which will not only reduce the dependence of countries on extracted raw hydrocarbon materials and coal but will also solve a number of issues to reduce the emission of pollutants into the atmosphere. The concept of the development of the energy sector adopted by most countries includes an increase in the share of nuclear energy in the generation of electricity, as well as the development of other alternative ways of generating energy, including hydrogen and thermonuclear energy [3,4].

One of the key concepts for the development of nuclear power, which is associated with an increase in the capacity of nuclear power plants, is the creation of modern types of nuclear reactors (high-temperature reactors, fast reactors, and modular reactors) with enhanced nuclear fuel burnup efficiencies, as well as high levels of resistance to the accumulation of radiation damage during operation and high levels of safety [5,6]. The key goal in creating these types of nuclear reactors is to amplify the efficiency of the use of nuclear fuel, with the possibility of replacing classical uranium fuel with plutonium fuel to develop a sustainable, closed fuel cycle for low-power or modular reactors, in addition to reducing costs by increasing the operating life and switching to high-temperature operating modes, with the possibility of carrying out work aimed at the production of hydrogen, which can later be used as a carbon-neutral fuel. At the same time, in the design of new-generation reactors (Gen IV), much attention is being paid to development and research work aimed at studying the possibilities of using new types of materials, including ceramics and high-entropy alloys, as candidate materials for the design of the core and the key components of a nuclear reactor, as well as their use as materials for fuel elements in the form of inert matrixes or fuel rod walls [7,8,9,10]. It should also be noted that when considering new types of structural materials, significant emphasis is placed on radiation resistance and the preservation of the stability of key parameters (thermal conductivity, strength, and embrittlement resistance) over extended periods of exposure to ionizing radiation. This is primarily due to the fact that during the operation of these materials, one should take into account the operating modes under which they will remain stable while also knowing the critical points at which the destabilization of the work may occur and finding ways to address these critical points.

### 1.2. Statement of the Reason for Studying Radiation Damage in Ceramics

Interest in studying radiation damage and establishing relationships between structural changes caused by irradiation and changes in optical, electronic, or thermophysical properties is primarily due to the need to accurately understand the mechanics of the accumulation of radiation damage in structural materials, as well as the need to identify critical factors that may have negative effects on the service lives of materials [11,12,13]. Furthermore, aside from their practical significance in examining the mechanisms of radiation damage, such studies hold fundamental importance. They enable the identification of the primary mechanisms governing the interaction between ionizing radiation and matter. Moreover, the experimental data obtained can be used to deduce the kinetics of the accumulation of radiation damage and its subsequent evolution within materials. It is worth noting that in this context, the duration of damage accumulation and its progression in the affected layer is a critical factor which is not only dependent on the type of irradiation (the ion type, energy, and irradiation mode) but also on the time frame [14,15]. As a rule, the most vulnerable point in a structural material subjected to external influences is the near-surface layer with a thickness of the order of several microns (10–20 µm) in which, due to the nature of the interaction of ionizing radiation, all structural changes caused by irradiation are concentrated. In the case of heavy ions, comparable to the fission fragments of nuclear fuel, the greatest changes associated with the interaction of accelerated ions along the trajectory of motion in the material occur at a depth of about 10–15 µm, in some cases up to 20 µm. Moreover, as was shown in [16,17,18], the depth to which ions penetrate exhibits a clear correlation with both the ion type and its energy. Structural alterations induced in the material throughout most of its depth can be attributed to the ionization losses incurred as incident ions interact with electron shells. In this case, the primary factors driving these alterations are the energy losses of incident ions during their interactions with the target crystal structure. Concerning high-energy ions, the difference between the ionization losses of incident ions during interactions with electron shells and nuclei is of several orders of magnitude, and the ionization energy loss of ions during interactions with electron shells plays a dominant role in structural changes [19,20]. At the same time, these changes in ceramics require a detailed study since most of the ceramics considered candidate materials are dielectrics in which alterations in the electron density and its distribution are irreversible in most cases; this is one of the key differences between ceramics and metals and alloys, in which a relaxation characteristic of the recovery of the electron density distribution after irradiation is observed. In recent years, there has been a significant research effort focused on investigating the connection between structural alterations and the electron density distribution. This interest primarily stems from the need to gain insights into the fundamental principles underlying the irradiation effect and the accumulation of radiation-induced damage in ceramics [21,22,23,24].

The key goal of this study is to establish a connection between structural distortions due to the accumulation of radiation damage and alterations in the optical and electronic properties of polycrystalline ceramics based on aluminum nitride which are exposed to heavy Xe^23+^ ion irradiation with varying fluences, taking into account models of the effects of single interactions and the effects of overlapping defective regions. The selection of ion types for irradiation is based on the ability to simulate radiation damage that is analogous to the effects of nuclear fuel fission fragments and their accumulation with varying irradiation fluences. The choice of the energy of the incident ions (no more than 230 MeV) is due to their ability to create radiation damage and accumulate in a near-surface layer with a thickness of 15–16 μm, which is typical for most near-surface layers exposed to external influences in the operation of structural materials in the cores of nuclear reactors or fuel-rod materials. In this work, considerable focus is placed on the study of the relationship between the structural characteristics and the optical (transmission, absorption, and changes in the band gap) properties of ceramics when the irradiation fluence is varied. At the same time, a comparative analysis of the observed changes makes it possible to obtain a number of new dependences in ceramics.

## 2. Experiment

### 2.1. The Object of Study

A polycrystalline aluminum-nitride-based ceramic (AlN) (CRYSTAL GmbH, Berlin, Germany). with a polycrystalline structure comprising a hexagonal crystal lattice (wurtzite type), the parameters a = 3.1149 Å and c = 4.9820 Å, and a density of 3.26 g/cm^3^ was chosen as the object of this study. The choice of this type of ceramic as a research subject was due to its great prospects for use as a material in the production of nuclear energy, in particular as a structural material in the first wall of a reactor or as an alternative to the materials used in the walls of fuel elements or classical zirconium alloys. Among several prospective ceramics, aluminum nitride (AlN) was identified as one of the best candidates for nuclear applications owing to its high tolerance to radiation damage [25,26,27]. In addition, single aluminum nitride (AlN) crystals are considered promising candidates for sensing units due to aluminum nitride’s excellent resistance to conditions with high temperatures and levels of irradiation [28,29].

The ceramic samples investigated to carry out studies related to the assessment of the effects of irradiation on alterations in optical and strength properties were 10 mm × 10 mm plates with a thickness of about 20 µm. This sample thickness was chosen considering the maximum depth of the ions’ paths in the material (15–17 µm) so that all observed changes in the properties assessed could be correlated with alterations induced by irradiation.

### 2.2. Setting the Goal and Objectives of this Study

The primary goal of this work was to study the influence of irradiation with heavy Xe^23+^ ions with a total energy of 230 MeV on variations in the structural, optical, and strength properties of AlN ceramics when the irradiation fluence varied in the range from 10^10^ to 5 × 10^13^ ion/cm^2^ [23,24]. The main objective of the study was to ascertain how changes in irradiation fluence are linked to alterations in ceramic properties and to elucidate the mechanisms responsible for the creation and subsequent development of point and vacancy defects, particularly of the *O_N_*–*V_Al_* type, within the damaged layer, all in relation to variations in the irradiation fluence.

### 2.3. Irradiation of Samples in Order to Simulate the Processes of Radiation Damage Accumulation

The accumulation of radiation damage was simulated to determine the kinetics of fluctuations in the structural and optical properties via the irradiation of the studied polycrystalline ceramic samples with heavy Xe^23+^ ions with a total energy of 230 MeV (1.75 MeV/nucleon) in a range of irradiation fluences from 10^10^ ion/cm^2^ to 5 × 10^13^ ion/cm^2^. The ion flux density did not exceed 10^9^ ion/cm^2^·s; this limit was chosen to prevent the target from overheating during long-term irradiation and the subsequent destabilization of radiation damage during thermal heating. The selection of the irradiation fluence range was due to the possibility of modeling single instances of radiation damage, as well as creating areas of overlap of defective regions due to the accumulation of radiation damage along the trajectory of the ions’ movement in the near-surface layers of the ceramic samples. In this case, an assessment of the effect of the overlap of structurally changed regions arising along the trajectory of ions in the material was conducted based on the following assumptions.

According to a number of theoretical and experimental works [30,31,32], the diameter of such a region formed along the trajectory of ions in a material is about 3–10 nm, depending on the type of incident ions and their energy. With the given diameters, the probability that two similar regions will be at a maximum approximate distance from each other (less than 5 nm) corresponds to an irradiation fluence above 5 × 10^11^ ion/cm^2^. In the case of irradiation fluences above 10^13^ ion/cm^2^, the effects of the so-called deep overlapping of structurally changed areas are observed, which can lead to a sharp deterioration in the properties of ceramics due to large changes in the distribution of electron density and the accumulation of structural deformation distortions in the damaged layer. A distinctive feature of this work is that the irradiation was performed with a small fluence change step to implement a complete analysis of the relationship between the structural and optical properties of the samples.

**Figure 1:**
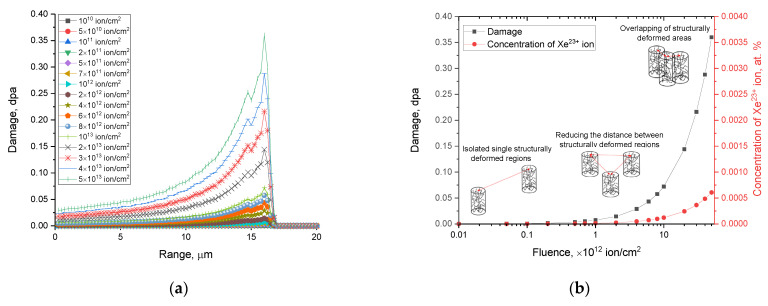
(**a**) The results of constructing the dependence of the atomic displacement value along the trajectory of ions in ceramics with variations in irradiation fluence; (**b**) the results of the assessment of the dependence of the variation in the values of atomic displacement and the concentration of implanted Xe^23+^ ions at the maximum depth of the free path (15 µm) in the near-surface layer.

It is also worth noting that in the irradiation fluence range of 10^10^–10^12^ ion/cm^2^, the displacements are less than 5 dpa each, and the greatest changes and, accordingly, an increase in the dpa, are observed at fluences above 10^12^ ion/cm^2^, which are characterized by the formation of the effects of overlapping areas of radiation damage along the trajectory of ions in the material (see data in Figure 1a). In this regard, the peak value of atomic displacement in the majority of ion trajectories is no more than 0.0003 dpa, and it sharply increases from 0.05 to 0.14 dpa in the region of 1–15 µm, with a maximum of 0.144 dpa at 16 µm, followed by a sharp decrease. This behavior of the atomic displacement value is typical for the dominance of the processes of the elastic collisions of incident ions with nuclei during the deceleration of ions in the material near their maximum trajectory.

Figure 1b reveals the results of estimating the dependence of variations in the values of atomic displacements and the concentration of the implanted Xe^23+^ at the maximum depth of travel in the near-surface layer, obtained via modeling using SRIM Pro 2013 program code. As is evident from the data presented, the maximum atomic displacement value is 0.35 dpa at a fluence of 5 × 10^13^ ion/cm^2^, while the concentration of the implanted Xe^23+^ is less than 0.0005 at. %. In this case, despite the nature of the implanted Xe^23+^ ions in the damaged layer of the ceramic sample, even at the maximum irradiation fluence in this series of experiments, it is not worth judging the possibility of the formation of gas-filled bubbles which lead to gas swelling in view of the low concentrations of Xe^23+^ ions. Thus, from the calculated indicators, we may deduce that the dominant contribution to the structural changes in the damaged layer will be made by the effects associated with ionization losses (fluctuations in the electron and optical densities), as well as structural distortions due to deformation stresses that occur at high irradiation fluences. The tabs in Figure 1b also show schematic representations of the isolated, structurally changed regions that formed, which appear along the trajectory of the ions’ motion in the ceramics depending on the irradiation fluence. When it comes to low fluences, these regions are positioned at significant separations from one another, and according to the simulation results, this does not result in a substantial occurrence or buildup of atomic displacements within the damaged layer. In the case in which the irradiation fluence reaches values characteristic of the convergence of these isolated, structurally changed regions due to an increase in irradiation density, increases in atomic displacement and the concentration dependence of the Xe^23+^ ions implanted in the surface layer are observed. At the highest levels of irradiation fluence, the effect of the overlap of these structurally changed regions leads to a sharp growth in the magnitude of atomic displacements, which is accompanied by an increase in the concentration of defective inclusions in the damaged layer.

### 2.4. Methods for Researching and Determining Structural and Optical Changes in Irradiated Ceramics

As the main methods for studying the effect of irradiation with heavy ions on changes in the properties of the near-surface layers of ceramics, non-destructive control methods, such as X-ray diffraction (the assessment of changes in structural parameters), optical UV-Vis spectroscopy (the determination of the influence of the accumulation of structural damage on the change in optical properties), and Raman spectroscopy (the determination of residual mechanical stresses in the damaged layer), were chosen. The choice of these research methods was due to the possibility of studying the kinetics of the accumulation of radiation damage kinetics in the structure of the damaged layer and to establish a connection between the accumulation of defects in the damaged layer and changes in the properties of the ceramics.

To measure the structural parameters, as well as their variations depending on the type of external influences, particularly the increase in the irradiation fluence, the X-ray diffraction method was used. A series of diffraction patterns reflecting these changes depending on the degree of radiation damage accumulated were obtained using a D8 Advance ECO X-ray diffractometer (Bruker, Berlin, Germany). The X-ray diffraction patterns were taken in Bragg–Brentano geometry in the angular range of 2θ = 30–80°, in increments of 0.03°, and with a spectrum acquisition time at a point of 1 s. The structural parameters were evaluated by comparing them with the reference values, and this was performed using DiffracEVA v.4.2 program code (Bruker, Berlin, Germany). The PDF-2 (2016) database was used to refine the phase composition and the parameters of the reference values.

The optical characteristics were assessed by processing the obtained optical transmission and absorption spectra in the range from 300 to 1000 nm. These spectra were obtained using a Specord-250 UV spectrophotometer (Jena Analytic, Jena, Germany). The band gap value (*E_g_*) was determined by constructing Tauc plots and conducting an analysis using Formula (1):

(1) $$\alpha =A{\left(h\nu -E_{g}\right)}^{1/2},$$

where *A* is a constant and *hν* is the photon energy.

The definition of the refractive index (*n^optical^*) was obtained using Formula (2):

(2) $$\frac{\left[{\left(n^{optical}\right)}^{2}-1\right]}{\left[{\left(n^{optical}\right)}^{2}+2\right]}=1-\sqrt{\frac{E_{g}}{20}},$$

Since the measurements were performed on samples with a thickness of 20 µm, comparable to the maximum depth of the ion path in ceramics, the changes in the band gap, as well as the refractive index, reflect the effect of the accumulation of radiation damage in the ceramics which was caused by irradiation.

Raman spectra reflecting the dynamics of the accumulation of residual mechanical stresses in the damaged layer were obtained using an Enspectr M532 spectrometer (Spectr-M LLC, Chernogolovka, Russia) operating at a wavelength of 532 nm. An analysis of the dynamics of the changes in residual stresses was executed via a comparative analysis of the position of the main spectral lines A_1_(TO), E_2_(high), and E_1_(TO), which are characteristic of AlN polycrystalline ceramics, as well as by measuring changes in the FWHM values of the spectral lines, which characterize the accumulation of amorphous inclusions in the damaged layer.

Hardness measurements were carried out using a MIKON Duroline-M1 microhardness tester (METKON instruments, Bursa, Turkey). A Vickers pyramid was used as an indenter. The measurements were carried out at various loads in order to establish changes in the values of hardness and softening at different depths. Measurements of the resistance of the ceramics to a single compression were carried out using a testing machine LFM-L 10 kN (Walter + BaiAG, Löningen, Switzerland).

A study of the morphological features resulting from structural alterations connected to the accumulation of deformation distortions in the damaged near-surface layer was carried out by analyzing 3D images of the surface. An AIST NT scanning probe microscope (AIST, Zelenograd, Russia) was used to construct 3D images via the atomic force microscopy method. The survey was carried out via the surface scanning method in semi-contact mode on an area measuring 10 µm × 10 µm.

## 3. Results and Discussion

### 3.1. Results of Changes in the Structural Characteristics of AlN Ceramics as a Result of Irradiation with Heavy Xe^23+^ Ions

**Figure 2:**
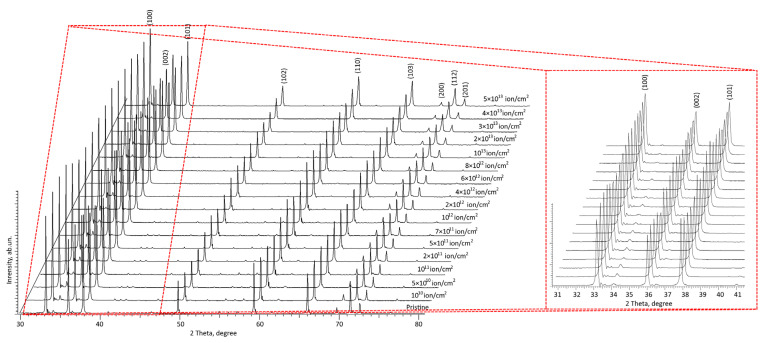
Results of measurements of X-ray diffraction patterns depending on the fluence of irradiation with heavy Xe^23+^ ions with variations in the irradiation fluence (red highlights the area of change in the main diffraction reflections in the region 2θ = 32–39°, reflecting structural changes resulting from irradiation).

Figure 2 shows the results of measurements of the X-ray diffraction patterns of the samples under study, performed in Bragg–Brentano geometry (2θ = 30–80°), reflecting the kinetics of changes in the structure of the ceramics depending on the irradiation fluence. Upon analyzing the presented X-ray diffraction patterns depending on the irradiation fluence, it was revealed that there were no changes linked to the formation of new reflections in the diffractogram or the appearance of the effect of reflection stratification, which indicates an absence of phase changes during the irradiation of the ceramics over the entire fluence range under study. The main observed alterations in the diffraction patterns presented are associated with insignificant variations in the intensity of the diffraction reflections, as well as their shift to a region of small angles at irradiation fluences above 10^12^ ion/cm^2^. In this case, the established changes indicate that the main processes associated with the accumulation of radiation damage in ceramics are due to structural deformation distortions, the type and nature of which depend on the irradiation fluence. At fluences below 10^12^ ion/cm^2^, the observed slight changes in the intensity of the main diffraction reflections, as well as their ratios, may be due to grain misorientation and textural effects that occur in the case of ionization processes in the damaged layer. Moreover, the absence of significant shifts of reflections relative to the position of the maxima in the initial state at fluences below 10^12^ ion/cm^2^ implies a high resistance of the crystal lattice of this type of ceramic to the ionization processes associated with the interaction of incident ions with electron shells. An analysis of changes in the intensities of diffraction reflections depending on the irradiation fluence showed that the most pronounced changes associated with a decrease in intensity, as well as the broadening of diffraction maxima, are observed for fluences above 5 × 10^12^ ion/cm^2^. The formation of an asymmetric shape of diffraction reflections, and a decrease in intensity, can be due to the effects of the accumulation of structural disorder in the damaged layer, as well as the formation of regions with high concentrations of stresses and strains. As the fluence increases, these structurally isolated regions approach each other. In this case, the resulting structural distortions within one isolated region can have a negative impact on the already existing structural distortions within a nearby region, thereby destabilizing the crystal structure. In this case, the resulting point defects and vacancies can begin to interact with each other, forming cluster or complex defects that have sufficiently high mobility in the damaged layer. The resulting deformation distortions, leading to the destabilization of the damaged layer at high densities, can lead to the appearance of structurally disordered regions characterized by the absence of any structural order (amorphous inclusions).

Moreover, the analysis of the obtained X-ray diffraction data for the studied ceramics irradiated with the maximum fluence (for this experiment) showed the presence of diffraction reflections that have only a distorted shape, which implies that these ceramics have a high level of resistance to amorphization processes as a result of irradiation in contrast to silicon nitride ceramics, for which the partial or complete amorphization of the damaged layer was observed at similar irradiation fluences [33]. Also, the absence of variations associated with the formation of new diffraction reflections for irradiated samples, which characterize polymorphic phase transformations, indicates the resistance of the ceramic structure to these processes, which makes them more promising in comparison with ZrO_2_ ceramics, for which these effects were established upon irradiation with heavy Kr and Xe ions [34,35].

One of the ways to assess the degree of influence of ionizing radiation on the crystal structure of a ceramic is to assess the deformation distortion of the crystal lattice, the nature of which indicates the resistance of a ceramic to radiation damage and also allows you to evaluate the kinetics of the accumulation of structural distortions and establish the main mechanisms of the deformation of the crystal structure. As a rule, the deformation distortions of the crystal structure in the case of a non-cubic type of crystal lattice are evaluated for each parameter separately, which makes it possible to establish not only the type of deformation distortion (tensile or compressive) but also the degree of distortion isotropy. In the case of an anisotropic distortion of the crystal lattice, the changes in the parameters will be uneven which, in turn, can result in the appearance of effects associated with the formation of metastable deformation inclusions in the structure.

**Figure 3:**
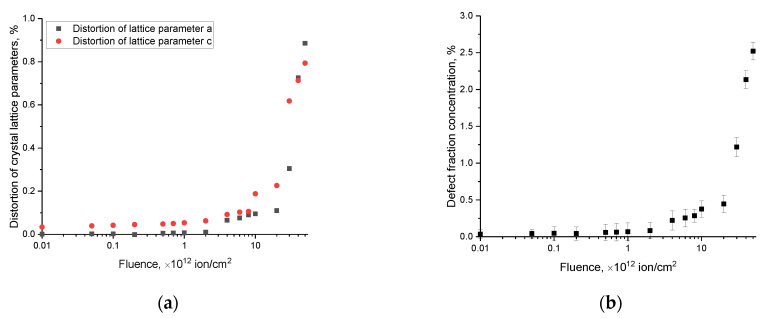
(**a**) The results of estimation of distortion of AlN crystal lattice parameters depending on irradiation fluence; (**b**) the results of calculating the concentration of the defective fraction in the damaged layers of the ceramic samples depending on the irradiation fluence.

Figure 3a shows the results of the estimation of the deformation distortions of the crystal lattice parameters *a* and *c*, characteristic of the hexagonal type, depending on the fluence of irradiation with heavy Xe^23+^ ions, which characterize the structural distortion, as well as the resistance of the crystal structure to deformation distortions. The general view of the observed changes in the deformation distortions of parameters *a* and *c* depending on the irradiation fluence indicates that they can be characterized by several stages. The first stage is typical for irradiation fluences of 10^10^–10^12^ ion/cm^2^ for which the observed changes correspond to small deformation distortions of the crystal lattice (less than 0.05%), while both parameters *a* and *c* change isotropically depending on the irradiation fluence (i.e., the difference in deformation distortions remains at the same level with an increasing irradiation fluence). With an increase in the irradiation fluence above 10^12^ ion/cm^2^, which is characterized by the formation of overlapping effects of structurally deformed regions, a more pronounced deformation of the parameter *c* is observed, while the deformation distortion of the parameter *a* is less pronounced. However, at fluences of 3 × 10^12^–5 × 10^13^ ion/cm^2^, the reverse picture of the change in deformation distortions is observed with the distortions of parameter *a* dominating. This difference can be explained by the appearance of the effect of overlapping defective regions at high irradiation fluences, leading to the anisotropic distortion of the crystal lattice. The anisotropic distortion of the crystal lattice parameters at irradiation fluences above 10^12^ ion/cm^2^ indicates an increase in structural deformations, the accumulation of which is accompanied by increases in various distorting factors, including the formation of vacancy complexes and cluster defects, the presence of which was confirmed in a number of works [36,37].

It should be noted that the positive values of distortions of the crystal lattice parameters indicate that the deformation distortion is due to swelling effects as a result of the accumulation of tensile stresses in the structure of the damaged layer. At the same time, the most pronounced changes in the crystal lattice parameters were observed for irradiation fluences above 10^12^ ion/cm^2^ at which, according to the data from a number of studies in the literature [38,39,40], there is an overlapping effect of local structurally deformed regions that arise along the trajectory of the incident ions within the crystal structure of the target material.

Based on changes in the structural parameters and the volume of the crystal lattice, the concentration of the defective fraction (*F_d_*) was calculated using the formula *F_d_ =* 1 − VcV, where *V* and *V_c_* are the crystal lattice volumes before and after irradiation. The results are shown in Figure 3b. The general view of the trend in changes in the concentration of the defective fraction corresponds to the trend in changes in the deformation distortions in the crystal structure, with a swift increase in concentration at fluences above 5 × 10^12^ ion/cm^2^, implying a cumulative effect, as well as the influence of the effect of the overlap of local structurally deformed regions, leading to an acceleration of the processes of the destruction of the crystalline structure of the damaged layer. The most pronounced deformation distortions, as is evident from the data presented, cause the accumulation of the defective fraction in the damaged layer and its exponential increase at the highest irradiation fluences. At the same time, deformation mechanisms are caused by tensile stresses and the subsequent swelling of the crystal lattice which, in turn, can lead to a deterioration in the strength characteristics of the material or the formation of hillocks on its surface due to the displacement of the deformed volume.

### 3.2. Study of the Effect of Irradiation on Changes in the Optical Properties of AlN Ceramics

One of the methods for assessing the effect of irradiation on the overall change in the properties of materials is the method of comparatively evaluating the optical transmission or absorption spectra of samples before and after external influences. As a rule, a change in an optical characteristic (transmission, optical density, or absorption) depending on external influences is associated with the accumulation of defective inclusions in the structure, as well as changes in the electron density associated with the redistribution of electrons on electron shells under the action of external forces.

**Figure 4:**
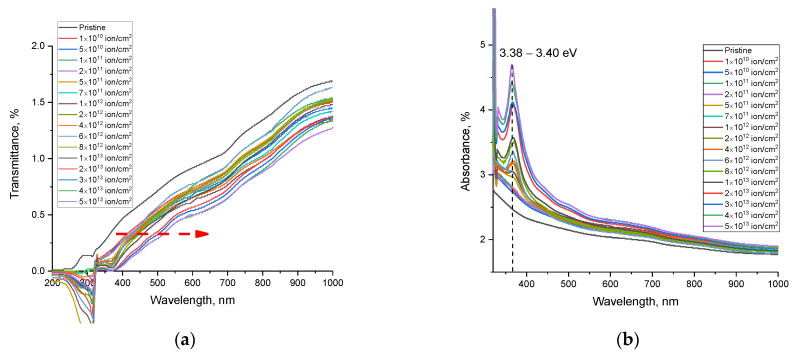
(**a**) The results of changes in the UV-Vis transmission (**a**) and absorption (**b**) spectra of AlN ceramics depending on the irradiation fluence (the arrow in figure (**a**) shows the shift in the transmission spectra, indicating a change in the fundamental absorption edge).

Figure 4a demonstrates the results of variations in the optical UV-Vis transmission spectra depending on the fluence of irradiation with heavy Xe^23+^ ions. The overall depiction of the observed transmission spectrum trends manifests as alterations in optical transmission within the 400 to 1000 nm range, encompassing both the visible spectrum and the near-infrared spectrum (above 780 nm). At the same time, for the irradiated samples, in addition to a change in transmittance in the measured wavelength range, there is also a shift in the fundamental absorption edge at the border of the ultraviolet and visible regions of 330–380 nm, with a shift of the edge toward the visible light region. The decrease in transmission intensity in the visible light and near-infrared ranges is due to an increase in phonon absorption associated with the accumulation of radiation damage. It should be noted that the greatest changes are observed at fluences of 10^10^–5 × 10^12^ ion/cm^2^, for which not only is a shift of the fundamental absorption edge observed but a decrease in the transmission intensity is also observed, the change in which indicates the formation of changes in the damaged layer linked to the formation of point defects, as well as alterations in the electron density and its distribution near the trajectories of the incident ions when they pass through the ceramic.

An analysis of the absorption spectra presented in Figure 4b made it possible to establish two characteristic types of variations related to alterations in the irradiation fluence. Firstly, for all the absorption spectra of the irradiated samples, an increase in the absorption intensity was observed over the entire measured range, which indicates a change in both the optical density characterizing the transmittance of the ceramics and an increase in the number of absorbing centers, leading to an increase in absorption and, as a result, a decrease in transmission.

An analysis of the dependences of the absorption spectra showed the formation of additional spectral absorption bands in the region of 3.38–3.40 eV at irradiation fluences of 5 × 10^11^ ion/cm^2^, which are characteristic of the formation of *O_N_*–*V_Al_* vacancy complexes. According [41,42], a similar absorption band at 3.35 eV, observed after reactor neutron irradiation at a dose of 10^16^ n/cm^2^ (*E* > 0.1 MeV), was designated an F-type center from the optical absorption and electron spin resonance. Later, F centers were considered some of the main point defects in AlN nanotubes [43]. Moreover, the intensity of the spectral absorption band for these vacancy complexes increases with a change in the irradiation fluence which, in turn, implies an elevation in the concentration of these *O_N_*–*V_Al_* complexes in the damaged layer. The presence of such inclusions in the form of *O_N_*–*V_Al_* is in good agreement with the data from a number of studies in the literature in which, as an explanation for the appearance of such complexes, they consider the mechanisms associated with the formation of defects and the subsequent recombination of donor–acceptor pairs associated with *V_Al_* vacancies and oxygen atoms replacing nitrogen atoms. The presence of oxygen in the compositions of ceramics is associated with the processes of manufacturing ceramics, alongside the presence of stabilizing additives in the form of Y_2_O_3_ compounds. Also, the change in the intensity of the spectral line at 3.38–3.40 eV, which characterizes the formation of *O_N_*–*V_Al_* vacancy complexes, can be considered an indicator of structural damage in ceramics during irradiation. At the same time, the absence of this spectral line at low irradiation fluences (below 5 × 10^11^ ion/cm^2^) indicates that the formation of these vacancy complexes occurs only when the distance between local structurally changed regions becomes very close.

**Figure 5:**
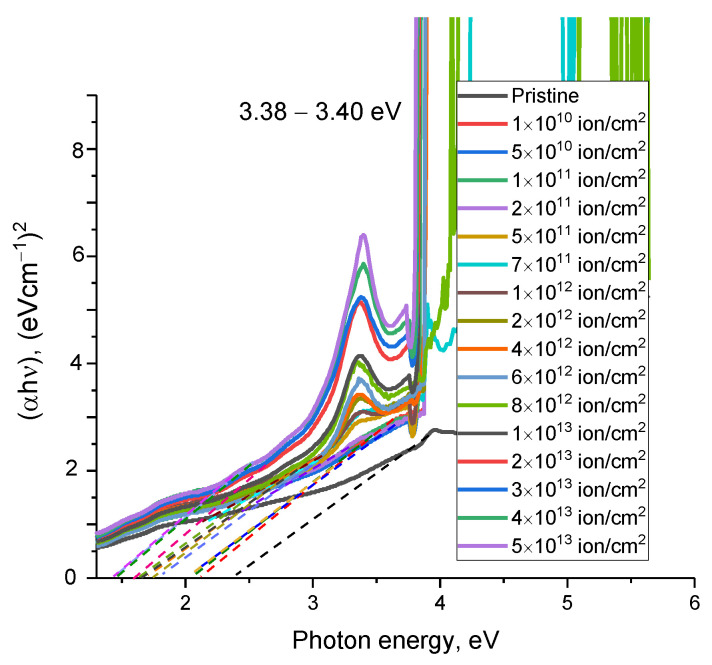
The results of the assessment of the changes in the band gaps of AlN ceramics as a result of irradiation, obtained by constructing Tauc plots.

A change in the band gap as a result of the accumulation of radiation damage with an increase in the irradiation fluence can lead to an increase in the effects associated with the ionization of the substance in the damaged layer since the ionization energy is directly dependent on the band gap (See Figure 5). As a result, the optical transmission spectra show a shift in the fundamental absorption edge to the region of long wavelengths, as well as a decrease in the transmittance of the ceramics in the entire measured range. In this case, the decrease in transmittance can also be explained by a change in the value of the refractive index, which has an inverse dependency on the band gap. An increase in the refractive index, in turn, indicates an increase in defects in the structure of a ceramic, an increase in the concentration of which occurs with an increase in the irradiation fluence and, as a consequence, the formation of structurally disordered regions in the structure.

The observed shift in the approximating curve, which determines the value of the band gap, indicates its decrease with an increase in the fluence of irradiation with heavy ions. Such effects, as shown in [44,45], are primarily due to the creation of localized states in the band gap which lead to changes in the electron and optical densities, respectively. The resulting high-energy photons can lead to the formation of electronic transitions between extended states in the valence band and the conduction band, which leads to a change in the band gap.

**Figure 6:**
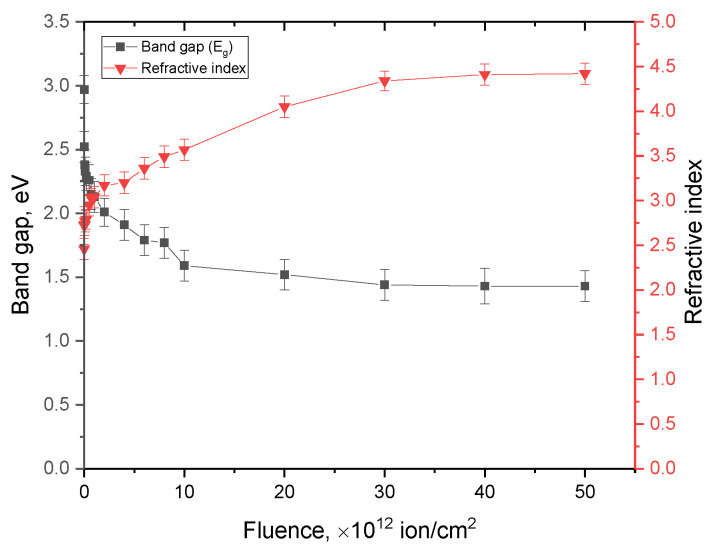
Dependences of the change in the band gap and the linear refractive index of AlN ceramics upon irradiation with heavy Xe^23+^ ions.

Figure 6 shows the dependences of the estimation of the change in the optical characteristics of the band gap and the linear refractive index of AlN ceramics upon irradiation with heavy Xe^23+^ ions.

The overall view of the presented changes in these values indicates a two-stage nature of the change depending on the irradiation fluence. At low irradiation fluences of 10^10^–10^12^ ion/cm^2^, there is a sharp decrease in the band gap and an increase in the linear refractive index, which characterizes an increase in the absorbing ability of ceramics. At fluences above 10^12^ ion/cm^2^, there is a slowdown in the trends of changes in these values, and in the case of irradiation fluences of 3 × 10^13^–5 × 10^13^ ion/cm^2^, there are practically no changes in these values. Such an effect can be explained by the saturation of changes in the electron and optical densities since at these irradiation fluences, as was found in the analysis of alterations in structural parameters, a steep increase in the deformation distortions of the structure is observed.

### 3.3. Determination of the Kinetics of the Formation of Residual Mechanical Stresses in the Damaged Layer of AlN Ceramics Using the Method of Raman Spectroscopy

**Figure 7:**
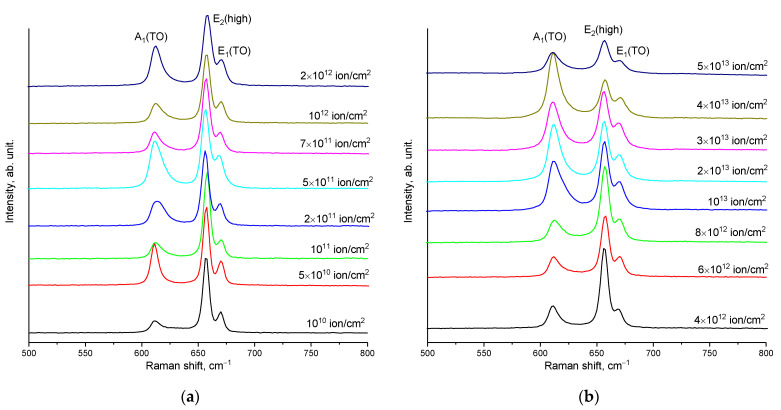
The results of measuring the Raman spectra of AlN ceramics irradiated with heavy Xe^23+^ ions, normalized to the maximum intensity with background subtraction in the wavelength range of 500–800 cm^−1^: (**a**) 10^10^–2 × 10^12^ ion/cm^2^; (**b**) 4 × 10^12^–5 × 10^13^ ion/cm^2^.

Figure 7 shows the results of measurements of the Raman spectra of the studied AlN ceramics depending on the fluence of irradiation with heavy Xe^23+^ ions. The spectra are presented as the dynamics of three main spectral lines, A_1_(TO), E_2_(high), and E_1_(TO), the changes in which characterize the kinetics of structural changes caused by external influences, as well as the formation of vacancy complexes of the *O_N_*–*V_Al_* type in the structure which arise during the accumulation of structural distortions in ceramics.

**Figure 8:**
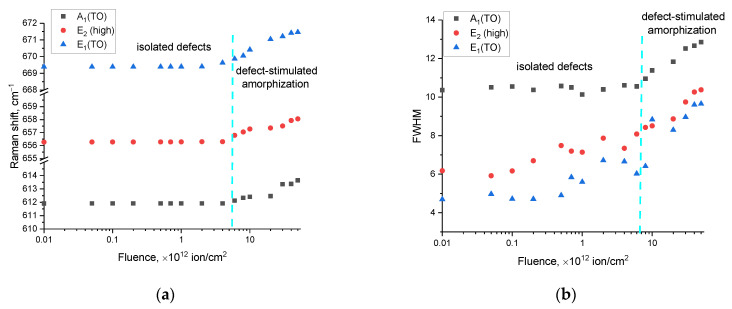
(**a**) The results of the evaluation of the change in the magnitude of the shift in the maximum of the spectral line; (**b**) the results of the evaluation of the change in the FWHM values of the spectral lines depending on the irradiation fluence.

By analyzing the kinetics of the changes in the Raman spectra of the main spectral lines characteristic of aluminum nitride, A_1_(TO), E_2_(high), and E_1_(TO), the following conclusions can be derived. First of all, the obtained spectra do not show the appearance of any new spectral lines, which indicates the preservation of the chemical order in the structures of the ceramics as a result of irradiation, as well as the absence of structural transformations caused by the accumulation of defects or ionization processes leading to a change in the electron density distribution. Secondly, the main observed changes in the spectral lines are due to two factors: the shift of the lines relative to the initial position of the maxima and the changes in their shape and linewidth (an increase in which characterizes the effect of amorphization as a result of the accumulation of radiation damage). The results of these fluctuations in two quantities (the shift in the maximum of the spectral lines and the FWHM) are shown in Figure 8.

An analysis of the shifts in the main spectral lines of the Raman spectra and the FWHM values shown in Figure 8 depending on the irradiation fluence indicate the cumulative effect of structural deformation distortions, which are most pronounced at a fluence above 10^12^ ion/cm^2^. In the case of low irradiation fluences, almost no alterations in the shifts of the spectral lines and FWHM values are observed, which implies a high level of resistance of the ceramic’s crystal structure to the occurrence of residual mechanical stresses in the damaged layer during the formation of isolated, structurally changed regions along the trajectory of the ions’ motion in the ceramic.

At fluences above 10^12^ ion/cm^2^, a positive increase in the displacement of the position of the spectral line maxima is observed which is characteristic of deformational distortions in the crystal lattice, and the dynamics of the displacement increase indicate a cumulative effect. At the same time, a similar trend is also observed for the FWHM value, an elevation in which indicates the formation of disordered, amorphous-like inclusions in the structure. Moreover, the obtained dependences of the changes in the values of the shifts of the spectral lines and the FWHM are in good agreement with the data on the deformation distortions of the crystal lattice measured using the X-ray diffraction method. This agreement indicates that the deformation mechanism of the structural changes is linked to the accumulation of crystal lattice distortions, alongside the formation of vacancy complexes of the *O_N_*–*V_Al_* type, the presence of which was established using Raman spectroscopy (line shift), as well as the analysis of optical absorption spectra. At the same time, it should be noted that the dynamics of changes in the shift values of the spectral lines, as well as the FWHM values for the three spectral lines A_1_(TO), E_2_(high), E_1_(TO), have the same trend in both the region characteristic of isolated defects and at fluences characteristic of the effects of overlapping defective regions and the formation of amorphous-like inclusions.

**Figure 9:**
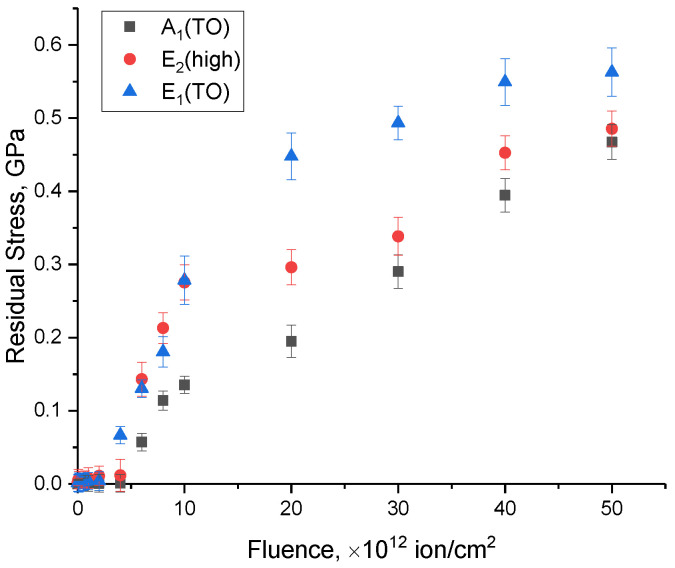
Results of the evaluation of the changes in the values of residual mechanical stresses in the damaged layers of ceramics depending on the irradiation fluence.

Based on changes in the positions of the spectral lines and their shifts, the values of residual mechanical stresses in the damaged layer were calculated, the presence of which is related to the processes of the transformation of the kinetic energy of incident ions during collisions with electrons and nuclei into thermal energy, as well as the subsequent processes of the deformation distortion of the crystal structure. The connection between the shifts in the spectral lines and the residual mechanical stresses (*σ_xx_*) was established in [46,47], according to which, using the difference in the positions of the maxima of the spectral lines (∆*ω*) and the expression ∆*ω* = *kσ_xx_*, where *k* = 3.7 cm^−1^/GPa is the strain coefficient, one can estimate the magnitudes of residual mechanical stresses in the damaged layer depending on the irradiation fluence. Moreover, the sign “+” or “−” in the calculations of these values characterize the type of residual mechanical stresses associated with tensile (“+”) or compressive stresses (“−”). The results of calculating the values of residual mechanical stresses for the three main spectral lines A_1_(TO), E_2_(high), E_1_(TO) are revealed in Figure 9, the general trend of which indicates that structural distortions in the structure of the damaged layer are due to tensile residual stresses resulting from the processes of the interaction of heavy ions with the crystal structure. At the same time, the most pronounced growth in the magnitudes of residual stresses in the damaged layer is observed in the range of 10^12^–10^13^ ion/cm^2^, in which there is an almost two- to threefold increase in the value of *σ_xx_*, while at fluences above 3 × 10^13^ ion/cm^2^, an increase in fluence leads to a slowdown in the trend of the accumulation of residual stresses in the structure of the damaged layer. The deceleration in the accumulation of *σ_xx_* can be rationalized via the presence of amorphous-like inclusions forming within the ceramic structure at these fluence levels. An elevation in their concentration results in the partial amorphization of the damaged layer, contributing to its deterioration. However, the concentration of these inclusions is low, as evidenced by the dynamics of the changes in the FWHM values of the Raman spectra and their intensities.

**Figure 10:**
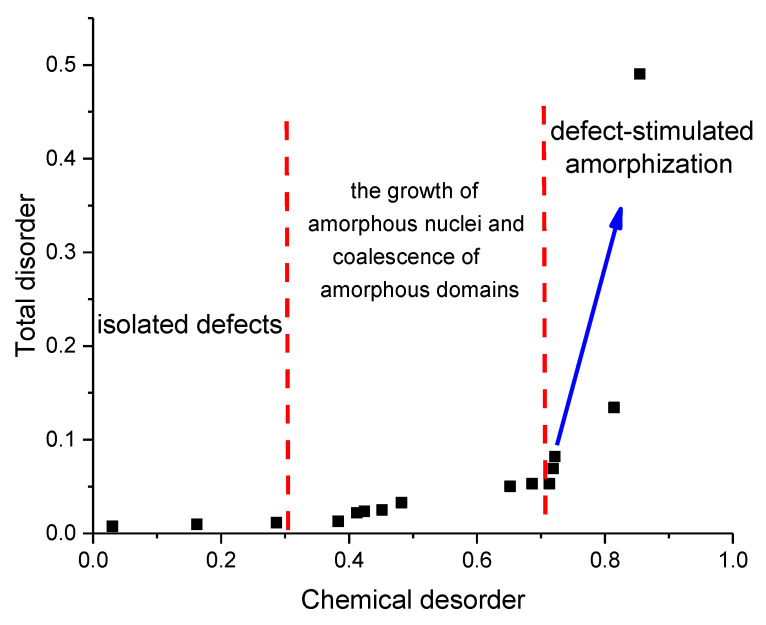
Ratios of the contributions of the general disorder and chemical disorder, calculated from the changes in the weight contributions of the spectral lines of the Raman spectra.

The parameters of total and chemical disorder caused by the irradiation of the crystal structure were determined by estimating the integral areas of the Raman spectra. An estimate of the total disorder was calculated using the ratios of the integral areas of the modes of the Raman spectral lines before and after irradiation. The chemical disorder was determined via the ratio of the integral areas and the intensities of the modes characteristic of Al–N chemical bonds. The results of a comparative analysis of the ratio of the contributions are shown in Figure 10. The general trend of the changes in the contributions of total and chemical disorder has a good correlation with the concentration of defects formed as a result of the interaction of incident ions with the crystal structure of the target in the damaged layer. The dependence itself can be divided into three characteristic stages associated with both the concentration dependence of the accumulation of defects and the consequences caused by this accumulation.

The initial phase exhibits minor alterations in both chemical and complete disorder, implying that locally isolated, structurally distorted defect regions are formed in the structure of the damaged layer, the concentration of which is low enough to affect the changes in the properties of the ceramics. Moreover, in this case, an increase in the density of these locally isolated defect regions leads to an increase in the chemical disorder caused by changes in Al–N chemical bonds as a result of their deformation or the formation of vacancy complexes.

The second stage is typical for fluences above 10^11^ ions/cm^2^ for which, in addition to an increase in the contribution of chemical disorder, an increase in the total disorder is also observed, an increase in which indicates a deformation distortion of the crystal structure as a result of an increase in the density of locally isolated regions, as well as the accumulation of point and vacancy defects. In this case, upon reaching the effect of the overlap of structurally distorted regions, an increase in chemical disorder as a result of an increase in the randomization of Al–N chemical bonds occurs, alongside the formation of vacancy complexes of the *O_N_*–*V_Al_* type, an increase in the concentration of which is clearly seen in the absorption spectra.

The third phase is marked by sudden increases in both total and chemical disorder which can trigger the onset of partial amorphization processes resulting from the accumulation of structural distortions and deformations in the damaged layer. In this case, locally deformed or structurally disordered regions can form in the structure, the presence of which leads to the amorphization of the damaged layer.

An essential factor influencing the determination of the potential application of ceramics as structural materials is the assessment of the stability of their strength and mechanical properties as a result of the accumulation of radiation damage in their near-surface layers. Furthermore, variations in the strength properties of ceramics are directly linked to the presence of defective inclusions that arise during high doses irradiation and to deformation distortions stemming from the accumulation of residual mechanical stresses.

**Figure 11:**
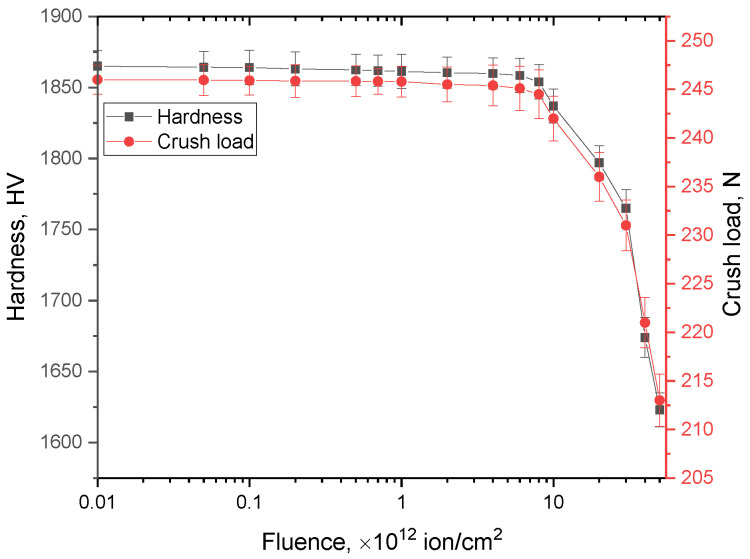
Results of the evaluation of changes in the strength characteristics (hardness and resistance to single compression) of the studied AlN ceramics depending on the irradiation fluence.

Figure 11 reveals the results of mechanical tests of the studied ceramics depending on the irradiation fluence, characterizing the changes in the strength properties of the ceramics in the case of the accumulation of structural changes in the damaged layer. The general trends in changes in the values of hardness and load resistance under a single compression are in good agreement with the data on changes in structural parameters linked to the accumulation of deformation distortions in the structure. The main changes in the strength characteristics are observed when the irradiation fluence reaches more than 10^12^ ion/cm^2^, which, as was previously shown, are characterized by the formation of structurally deformed regions and residual mechanical stresses linked to the cumulative effect, alongside the formation of overlapping defect regions.

**Figure 12:**
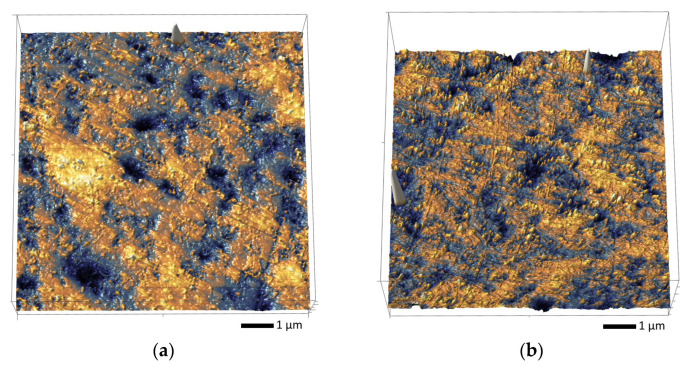

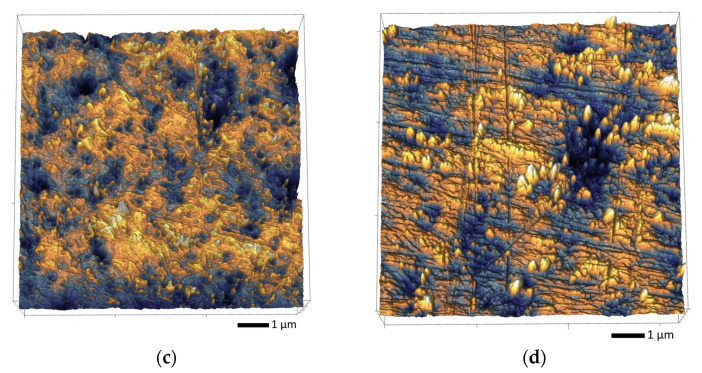
Three-dimensional reconstructions of ceramic surface morphology before and after irradiation with Xe^23+^ heavy ions: (**a**) pristine sample; (**b**) 10^12^ ion/cm^2^; (**c**) 10^13^ ion/cm^2^; (**d**) 5 × 10^13^ ion/cm^2^.

Figure 12 shows the results of the assessment of changes in the surface morphology of the AlN ceramics before and after irradiation with heavy Xe^23+^ ions at different fluences. The data are presented as reconstructed 3D images of the surfaces, which were obtained using the atomic force microscopy method.

An analysis of morphological features obtained by constructing 3D images of the ceramics’ surface profiles showed the formation of hillock-like inclusions on the surfaces of the irradiated ceramics; the number of inclusions increases at high irradiation fluences. In irradiated ceramic samples, the formation of hillocks, as shown in a number of works, is associated with the displacement of the surface volume in places of the accumulation of deformation distortions, which leads to the formation of spherical inclusions, the size of which can vary from several to hundreds of nanometers. Moreover, the densities of such inclusions and their sizes have pronounced dependences not only on the irradiation fluence but also, according to data from the literature, on the energy and type of incident ions. At the same time, it should be noted that at the maximum irradiation fluence, an increase in the size of the hillocks is observed, as well as the formation of small agglomerates from them which, in turn, is consistent with a sharp increase in deformation distortions in the damaged layer and the accumulation of residual mechanical stresses in the crystal structure, leading to its swelling.

Thus, it can be concluded that the accumulation of deformation distortions and residual mechanical stresses in the damaged layer of a ceramic results in the displacement of the deformed volume to the surface, resulting in the formation of pyramidal or spherical inclusions, the density of which increases with the irradiation fluence.

### 3.4. Establishing the Relationship between Structural Distortions and Changes in the Optical and Strength Properties of Ceramics under Irradiation with Heavy Ions

**Figure 13:**
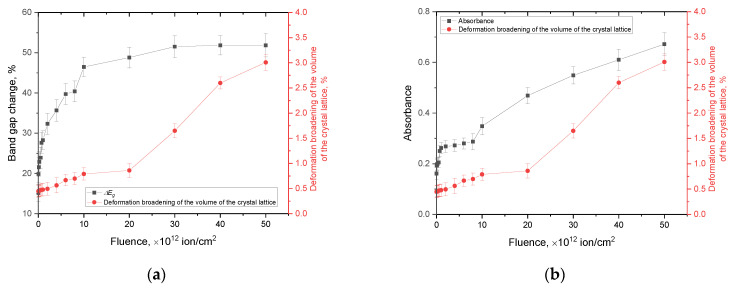
Comparative analysis of structural distortions and optical characteristics: (**a**) deformation via the volumetric swelling of the crystal lattice vs. changes in the band gap; (**b**) deformation via the volumetric swelling of the crystal lattice vs. changes in the optical density (absorbance was measured in the range of 450–550 nm).

Figure 13 demonstrates the results of a comparative analysis of fluctuations in the optical characteristics and structural distortions of the crystal lattice (volume swelling), reflecting the effects of irradiation on these characteristics. This analysis is presented in order to establish a relationship between changes associated with the accumulation of deformation distortions in a structure and its optical properties (the band gap and absorbance).

**Figure 14:**
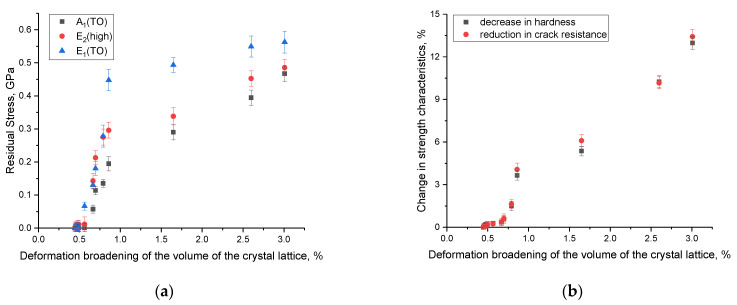
(**a**) Comparative analysis of the deformational volumetric swelling of the crystal lattice and residual mechanical stresses; (**b**) comparative analysis of deformation via the volumetric swelling of the crystal lattice and changes in its strength characteristics.

Figure 14 presents the data from a comparative analysis of the values of the volumetric swelling deformation of the crystal lattice and residual mechanical stresses, as well as changes in its strength properties as a result of the structural degradation of the damaged layer.

By analyzing the obtained dependences of alterations in the optical, structural, and mechanical properties of the AlN ceramics on the irradiation fluence, we can draw the following conclusions. At low irradiation fluences, which are characteristic of the formation of isolated, structurally deformed regions, ionization losses play a dominant role, leading to changes in the electron density due to the formation of localized states, as well as vacancy complexes, the presence of which results in the appearance of additional electronic transitions. Moreover, at low irradiation fluences, structural distortions of the crystal lattice practically do not manifest themselves, which indicates the material’s resistance to a low concentration of defective inclusions and the formation of isolated deformation distortions.

At fluences above 10^12^ ion/cm^2^, the dominant role is played by tensile residual mechanical stresses resulting from the effect of overlapping structurally deformed regions, as well as an increase in the deformation distortions of the crystal lattice. In this case, there is practically no change in the optical characteristics, particularly changes in the band gap at high irradiation fluences, while deformation distortions prevail under high doses of irradiation.

When comparing the deformation swelling of the crystal lattice and optical density data, a good correlation of these values is established, indicating the cumulative effect of deformation structural distortions that affect both the structural characteristics and the absorbing ability of ceramics. However, it should be noted that the linear nature of the change in the optical density (absorbance) with an increase in the irradiation fluence above 5 × 10^12^ ion/cm^2^ indicates not only the cumulative effect of structural deformations that change the absorbing ability of ceramics due to the formation of defective inclusions and vacancy complexes but also a direct correlation between the absorption capacity and the deformation distortion of the crystal structure. In this case, the obtained dependences of the change in optical density are in good agreement with data from a number of studies in the literature in which the change in this value characterizes the relationship between structural distortions and the absorbing ability of a material [48,49,50].

The dependences of changes in the values of residual mechanical stresses on the value of the deformation swelling of the crystal lattice presented in Figure 14a are in good agreement with each other, which is expressed in the cumulative effect of structural distortions, accompanied by an increase in the contribution of residual mechanical stresses in the damaged layer. At the same time, the most pronounced correlations of these values are observed at fluences above 5 × 10^12^ ion/cm^2^ when an overlap of structurally deformed regions is observed in the structure, leading to a sharp increase in the deformation of the crystal lattice.

The dependences of the degradation of strength characteristics on the deformation of the crystal structure determined in Figure 14b also have a good correlation with each other, indicating that the greatest influence on softening is made by the accumulation of residual mechanical stresses and deformation distortions. At the same time, in the case of the dominance of ionization effects associated with changes in the optical characteristics of ceramics at low irradiation fluences, changes in strength characteristics are practically not observed.

It is important to highlight that the analysis of the structural parameters and changes in the Raman spectra for AlN ceramics depending on the irradiation fluence did not reveal a high concentration of amorphous inclusions leading to the complete amorphization of the damaged layer, which is observed for Si_3_N_4_ ceramics, indicating a greater structural resistance of aluminum nitride to the accumulation of radiation damage. One of the explanations for such differences for the two classes of nitride ceramics may be that in the case of aluminum nitride, the crystal structure of the initial sample is represented by a single phase (hexagonal-like wurtzite), while most silicon nitride ceramics are a mixture of two phases, α and β. Hence, the accelerated amorphization processes in silicon nitride and the creation of latent tracks may be attributed to the impact of polymorphic transformations of the α↔β variety. Typically, these transformations coincide with a destructive alteration in the volume of the crystal lattice and an escalation of deformation distortions throughout phase transformations. In the case of the AlN ceramics, such effects were not observed, even with high doses of irradiation, and all the structural changes in the crystal lattice were associated with its deformation distortion and swelling.

## 4. Conclusions

In this work, the results of studies on the effects of irradiation with heavy Xe^22+^ ions on changes in the structural, optical, and mechanical properties of AlN ceramics were obtained. X-ray diffraction, UV-Vis spectroscopy, atomic force microscopy, Raman spectroscopy, and measurements of mechanical properties (indentation and single compression) were used as the main methods of analysis.

During the studies conducted, it was found that at low irradiation fluences (10^10^–10^12^ ion/cm^2^) with heavy Xe^22+^ ions, changes in the optical characteristics associated with changes in electron density distribution were more pronounced. At fluences above 10^12^ ion/cm^2^, the dominant role was played by deformation distortions of the crystal lattice associated with the accumulation of residual mechanical stresses and the effect of overlapping local structurally deformed regions.

During an analysis of the changes in mechanical strength properties depending on the irradiation fluence, it was found that the most pronounced changes occurred at irradiation fluences above 5 × 10^12^ ion/cm^2^ and consisted of a softening of the damaged layer and the deterioration of crack resistance.

The established connection between deformation distortions and changes in the optical and mechanical characteristics of ceramics can later be used to determine the optimal operating modes for these types of ceramics, as well as to justify the choice of types of structural materials for new-generation reactors. Certain relationships between the accumulation of structural distortions that occur along the trajectory of ions in the material and the formation of an absorption band characteristic of *O_N_–V_Al_* vacancy complexes can later be used for the identification of structural damage to ceramics upon irradiation with heavy ions.

## Data Availability

Not applicable.
